# Platelet-Rich Plasma Extract Derived from Animals Shows Potential in Promoting Wound Healing and Suppressing Inflammatory Response in Skin Cells

**DOI:** 10.3390/cells14070526

**Published:** 2025-04-01

**Authors:** Zheng-Qi Wang, Queenie Wing-Sze Lai, Xiong Gao, Qi-Yun Wu, Tina Ting-Xia Dong, Karl Wah-Keung Tsim

**Affiliations:** 1 Center for Chinese Medicine, Division of Life Science, The Hong Kong University of Science and Technology, Clear Water Bay, Kowloon, Hong Kong 999077, China; 2 Shenzhen Key Laboratory of Edible and Medicinal Bioresources, HKUST Shenzhen Research Institute, Shenzhen 518057, China

**Keywords:** deer blood, platelet-rich plasma, inflammatory, skin regeneration

## Abstract

Platelet-rich plasma (PRP) is an extract enriched with growth factors that facilitate skin regeneration and rejuvenation. Here, the functionalities of PRP derived from various animal sources have been investigated and compared, focusing on its potential therapeutic applications in skin regeneration. Total antioxidant capacity, wound closure, and melanin content in cultured keratinocytes were used to evaluate the efficacy of different animal PRP sources. The PRP derived from deer exhibited the highest performance and was selected for subsequent proteomic and metabolomic analyses. Our findings indicate that deer blood is an optimal source of animal-derived PRP, demonstrating significant properties in promoting wound healing, anti-inflammatory responses, and skin regeneration. This identified PRP from deer sources can be developed as a safe and effective product for skin rejuvenation and regeneration.

## 1. Introduction

Skin is essential for maintaining homeostasis by protecting the body from the external environment. Skin acts as a barrier that prevents harmful microbes and chemicals from entering the body and blocking harmful ultraviolet (UV) radiation from the sunlight [1]. The stratum corneum serves as a heterogeneous barrier that protects against dryness and environmental damage while preserving moisture. When the skin barrier is compromised, it can lead to increased trans-epidermal water loss and decreased hydration, resulting in wounds and potentially skin carcinogenesis over time. Skin aging is also a major concern, occurring naturally and being exacerbated by environmental factors [2,3]. As a result, recent aesthetic research has aimed at finding ways to reduce visible signs of aging by addressing both internal and external factors [4].

Platelet-rich plasma (PRP) refers to a plasma fraction concentrate rich in platelets obtained after centrifugation of whole blood [5]. It includes a variety of growth factors [6,7]. The elevated growth factors and fibrinogen can significantly enhance tissue regeneration and wound healing [8,9]. Recently, the application of PRP in dermatology has drawn great interest from industries [10,11]. In facial rejuvenation, the injection of PRP has shown positive effects on wrinkles, elasticity, and striae dispense, as well as application in plastic surgery [12,13,14]. Usage of PRP has also been reported in scar repair [15] and melasma regression [16]. However, most of the works on PRP are focused on autologous PRP or allogeneic PRP of the same species, and there are few reports on heterologous PRP, which greatly limits the application in clinics. Indeed, heterologous dogs’ PRP facilitated wound healing in rabbits, and there were no side effects observed [17].

The family *Cervidae*, commonly known as the deer family, comprises 23 genera and 47 species. The antlers from non-ossifying young male deer are being used as traditional Chinese medicine (TCM); the species includes *Cervus nippon* Temminck (red deer) and *C. elaphus* Linnaeus (sika deer). In addition, the blood of deer could also be used as TCM products. The earliest record of the medicinal properties of deer blood in China can be found in “Qianjin Yifang” by Sun Simiao in the Tang Dynasty (AD 581-682) [18]. Similar to humans, deer blood is rich in various crucial growth factors that have demonstrated anti-inflammatory and anti-aging properties. The traditional usages of deer blood in TCM include the treatment of anemia, fatigue, and weakness [19]. The usages of blood-derived products from deer, e.g., peptides, have been reported to have various therapeutic effects, including improving cognitive function and promoting wound healing [18,20,21].

PRP derived from animal sources may be highly effective in enhancing wound healing and facial rejuvenation. In searching for active non-human PRP, different animal blood sources were screened for their functionalities in anti-oxidation, wound healing, and melanin content in skin cells. The best source of PRP deriving from deer was identified, in accordance with its functions and safety. This research offers a fresh theoretical foundation for creating PRP from non-human sources as treatments for skin regeneration and rejuvenation, as well as in systematically determining the composition and function of deer-derived PRP, laying the foundation for the standardization and large-scale production of PRP.

## 2. Materials and Methods

### 2.1. Materials

Dulbecco’s modified Eagle medium (DMEM, Cat# 11965092) and fetal bovine serum (FBS, Cat# 16000044) were procured from Thermo Fisher Scientific (Waltham, MA, USA). Antibodies targeting NF-κB p65 (Rabbit mAb #8242) and filaggrin (Mouse mAb #sc-66192) were acquired from Cell Signaling Technology (Danvers, MA, USA) and Santa Cruz Biotechnology (Santa Cruz, CA, USA), respectively. RNAzol^®^ RT reagent (R4533), dimethyl sulfoxide (DMSO, D2650), and protease inhibitors (P8340) were supplied by Sigma-Aldrich (St. Louis, MO, USA). Commercial ELISA kits for VEGF (ab100662), FGF (ab99979), IGF (ab100545), and EGF (ab100510) were obtained from Abcam (Cambridge, UK).

### 2.2. PRP Isolation and Processing

Anticoagulated whole blood (3.2% sodium citrate) from unspecified animal donors was processed by centrifugation (2000× *g*, 15 min, 22 °C) to isolate PRP. The supernatant underwent three cycles of freezing (−80 °C, 1 h) and thawing (37 °C, 10 min) to lyse platelets and liberate growth factors [7]. UV-C irradiation (254 nm, 30 min, 15 W) ensured sterility prior to lyophilization. The resultant PRP extract was stored in light-protected vials at −20 °C.

### 2.3. Cell Culture

Human keratinocytes (HaCaT, ATCC^®^ PCS-200-011), murine melanocytes (Melan-A, a gift from Prof. Mingfu Wang), and B16F10 melanoma cells (CRL-6475™) were propagated in DMEM containing 10% FBS, 100 U/mL penicillin, and 100 μg/mL streptomycin. Cultures were sustained at 37 °C under 5% CO_2_, with media replenished every 48 h.

### 2.4. Growth Factor Quantification

ELISA-based detection of VEGF, FGF, IGF, and EGF in human and deer PRP followed established protocols [22]. Samples (100 μL) were incubated in BSA-blocked 96-well plates (30 min, 25 °C) for 2 h, followed by sequential addition of biotinylated detection antibodies (1:1000, 2 h) and streptavidin-HRP (1:5000, 1 h). The reactions were terminated with 2 N H_2_SO_4_, and absorbance at 450 nm was quantified (Multiskan™ FC).

### 2.5. Cell Viability Test

To assess the cytotoxicity of PRP_deer_ [23], 1 × 10^5^ cells/well (Melan-A, B16F10) were seeded in 96-well plates. After 24 h adhesion, the cells were exposed to PRP_deer_ (0–50 mg/mL) in DMEM for 24 h. After that, 100 μL of MTT (5 mg/mL) was added per well, incubated for 4 h at 37 °C, and formazan crystals dissolved in the same volume of DMSO. Absorbance at 570 nm was measured (SpectraMax^®^ i3x).

### 2.6. Melanogenesis Assay

Cultured Melan-A and B16F10 cells (1 × 10⁵ cells/well, 2 mL) were seeded onto 6-well plates overnight and then treated with PRP_deer_ (1, 2, 5, 10, 15 mg/mL) or vitamin C (a positive control at 1 mM) for 48 h. Melanin content was spectrophotometrically quantified at 405 nm based on a published paper [24].

### 2.7. AMES Bacterial Reverse Mutation Test

The Ames test (OECD 471) was employed based on published paper [25] to assess PRP_deer_ genotoxicity using five *Salmonella typhimurium* strains (TA98, TA100, TA1535, TA1537, TA102) under metabolic activation (S9 mix) and non-activation conditions. Mutagenic potential was defined as ≥2-fold increase in revertant colonies versus controls (SGS Life Science Services, ON, Canada).

### 2.8. Xenogenic Immune Test

Female BALB/c mice (6–8 weeks, 18–22 g) were randomized into five cohorts (*n* = 10): saline control, BSA (100 mg/kg), and PRP_deer_ (50/100/200 mg/kg). The procedures were approved by the Animal Ethics Committee of the Hong Kong University of Science and Technology (HKUST) (Reference No. PRP/073/20FX). Subcutaneous injections (0.12 mL) were administered weekly for four weeks. Post-euthanasia serum IgG, IgM, and C3a levels were measured by ELISA.

### 2.9. Wound Closure Assay

HaCaT cells at 3 × 10^5^ cells/well were seeded onto 6-well plates, and serum-starved overnight. Confluent HaCaT monolayers were scratched with sterile pipette tips, and treated with PRP_deer_ (1, 2, 5, 10, 15 mg/mL) or VEGF (20 ng/mL) in serum-free medium. Wound closure was monitored at 0 h (A_t0_) and 20 h (A_t20_)) using a Zeiss Axio Observer 7 microscope. T-Scratch software (version 3.29.1) calculated recovery rates as follows: recovery (%) = (A_t0_–A_t20_) × 100%/A_t0_.

### 2.10. DNA Transfection and Luciferase Assay

Inflammatory stimulation was initiated by treating murine macrophage RAW264.7 cells with lipopolysaccharide (LPS, 100 ng/mL) for 20 h at 37 °C in a humidified 5% CO_2_ incubator. Parallel experiments utilizing human HaCaT keratinocytes (1 × 10^5^ cells/well, 24-well plates) involved treatment with recombinant human TNF-α (20 ng/mL) for 6 h, with simultaneous administration of varying concentrations (1, 5, 10 mg/mL) of PRP_deer._ Pharmacological controls included dexamethasone (10 nM) dissolved in DMSO (final concentration < 0.1%).

Following treatments, cellular lysis was performed using lysis buffer. The homogenized lysates underwent centrifugation using a refrigerated Eppendorf 5424R microcentrifuge. Clarified supernatants (75 µL aliquots) were arrayed in black-walled 96-well microplates for luminescence analysis. Bioluminescent signals were quantified using a GloMax^®^ 96 Microplate Luminometer with 1s integration time following 5 min thermal equilibration. Signal normalization employed total protein concentrations determined via micro-BCA assay (Waltham, MA, USA; catalog no. 23235).

For pFLG2-eGFP transfected cell cohorts, fluorescence quantification was conducted using a microplate reader equipped with SoftMax^®^ Pro 7.1 software. Excitation at 488 nm (5 nm bandwidth) and emission detection at 509 nm (8 nm bandwidth) were performed using optimal photomultiplier gain settings determined by auto-ranging controls. Protein normalization utilized the Bradford method. All experimental conditions included triplicate technical replicates with three biological repeats, randomized across plate quadrants to control for positional effects.

### 2.11. RNA Extraction and Reverse Transcription

Inflammatory stimulation was initiated by treating murine macrophage RAW264.7 cells with lipopolysaccharide (LPS, 100 ng/mL) for 20 h in a cell incubator. Parallel experiments utilizing human HaCaT keratinocytes (1 × 10^5^ cells/well, 24-well plates) involved treatment with recombinant human TNF-α (20 ng/mL) for 6 h, with simultaneous administration of varying concentrations (1, 5, 10 mg/mL) of PRP_deer._ Pharmacological controls included dexamethasone (10 nM) dissolved in DMSO (final concentration < 0.1%).

Post-treatment, adherent cells were washed thrice with PBS and mechanically detached using cell scrapers. Total RNA isolation was performed using RNAzol^®^ RT reagent, with a modified monophasic phenol-guanidine isothiocyanate protocol. Cell lysates were homogenized in 500 μL RNAzol^®^ RT, followed by phase separation with 100 μL chloroform in nuclease-free polypropylene tubes.

RNA precipitation was achieved by mixing the aqueous phase with 250 μL 70% ethanol (*v/v*). Subsequent washing steps employed 75% ethanol (*v/v*) with vortex agitation at 1500 rpm for 10 s, followed by air-drying under laminar flow for 5 min. Purified RNA pellets were resuspended in 20 μL nuclease-free water and quantified via NanoDrop 2000c spectrophotometer (Thermo Fisher Scientific, Waltham, MA, USA) with dual-wavelength analysis (A260/A280: 1.8–2.1). Reverse transcription utilized the PrimeScript™ RT Master Mix in 10 μL reactions containing 5 ng RNA template.

### 2.12. Immunofluorescent Staining

HaCaT keratinocytes were cultured on #1.5 thickness glass coverslips pre-coated with poly-L-lysine (0.01% *w/v*) in 12-well plates at an initial density of 5 × 10^4^ cells/mL. Post-treatment, cells underwent sequential fixation with freshly prepared 4% paraformaldehyde in PBS for 10 min at 22 °C, followed by three washes with Tris-buffered saline (TBS).

Permeabilization and blocking were simultaneously performed using TBST buffer (TBS containing 5% BSA and 0.1% Triton X-100) for 120 min at 22 °C under orbital shaking (50 rpm). Primary antibodies were applied in humidity chambers at specified dilutions: rabbit monoclonal anti-NF-κB p65 (Cell Signaling 8242S; 1:200) and mouse monoclonal anti-filaggrin (Santa Cruz sc-66192; 1:50), with overnight incubation at 4 °C on a thermoelectric cooling plate.

Secondary detection employed spectrally distinct Alexa Fluor^®^ conjugates: donkey anti-mouse IgG-Alexa 488 (Abcam ab150105; 1:200) and donkey anti-rabbit IgG-Alexa 647 (Abcam ab150075; 1:200) dissolved in antibody stabilization buffer, incubated for 135 min at 22 °C under light-protected conditions. Nuclei were counterstained with ProLong™ Gold Antifade Mountant containing DAPI, cured for 24 h at 4 °C before imaging.

Confocal microscopy was performed on a Leica SP8 STED system (LAS X 3.7.4) equipped with HyD detectors and 63× oil immersion objectives. Sequential acquisition parameters included the following: 405 nm diode (DAPI), 488 nm Argon (Alexa 488), and 638 nm HeNe (Alexa 647) lasers with 5% power, 0.8 Airy unit pinhole, and 4× line averaging. Z-stacks (0.3 μm steps) were processed using LAS X 3.7.4 software with deconvolution algorithms.

### 2.13. Proteomics and Untargeted Metabolomics Analyses

The analyses of proteomics and untargeted metabolomics were performed by Shanghai Applied Protein Technology (Shanghai, China). The procedure of proteomics was consistent with a previous study [26], and the metabolomics was following the protocol established by [27].

### 2.14. Statistical Analysis

GraphPad Prism 8.0.1 was used here to conduct the data analysis. Statistical significance (* *p* < 0.05, ** *p* < 0.01, *** *p* < 0.001) was determined via one-way ANOVA with Tukey’s test.

## 3. Results

### 3.1. The Non-Human Sources of PRP

**Figure 1:**
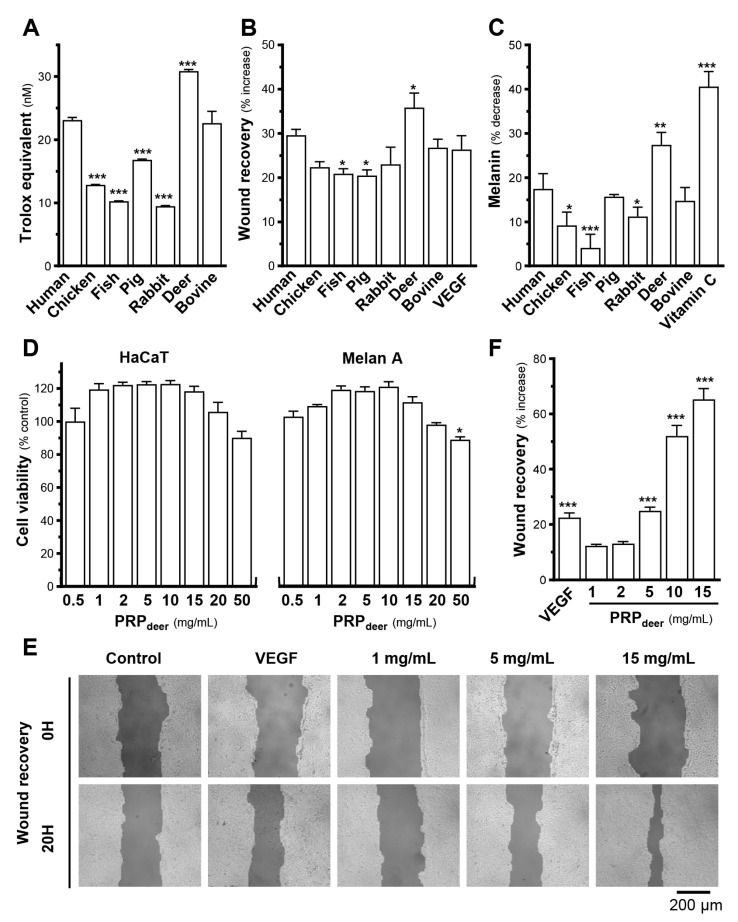
PRP_deer_ shows better effectiveness than other sources of PRP samples. Different animal sources, all at 5 mg/mL of PRP, were subjected to various assays of skin function A–C. (**A**) The anti-oxidation ability of different PRPs was determined by a commercial kit. The data are expressed as trolox equivalents. (**B**) Confluent HaCaT monolayers were scratched with sterile pipette tips; images illustrating wound healing were taken at 0 h (A_t0_) and 20 h (A_t20_). Quantitation of migration was calibrated as the following formula: (A_t0_–A_t20_) × 100%/A_t0_. (**C**) Different PRPs (5 mg/mL) were applied onto cultured melanocyte (Melan-A) cells for 48 h, and a melanogenesis assay was determined. (**D**) Different concentrations (0.5–50 mg/mL) of PRP_deer_ were applied onto cultured HaCaT and Melan A cells for 48 h, and an MTT assay was determined. (**E**) Cultured HaCaT cells created a wound; PRP_deer_ (1–15 mg/mL) was applied for 20 h. Images illustrating wound healing were taken at 0 h (A_t0_) and 20 h (A_t20_). (**F**) Quantitation of migration was calibrated. The values in mean ± SEM., *n* = 8. * *p* < 0.05; ** *p* < 0.01; *** *p* < 0.001 compared with corresponding human or control.

Different sources of PRP products, i.e., human, fish, chick, rabbit, pig, deer, and bovine, were chosen for comparison of their efficacies. Biochemical assays were developed and performed to test their activities relevant to the skincare functions. Firstly, antioxidant activity was compared as a screening indicator in these PRP samples. The results showed that PRP from deer blood (PRP_deer_) showed the best antioxidant capacity, with 30.9 nmol trolox equivalent (Figure 1A). The human sample (PRP_human_) showed an antioxidant capacity of 23.2 nmol trolox equivalent. Their ranking in terms of antioxidative activities was followed by bovine (PRP_bovine_), pig (PRP_pig_), chicken (PRP_chicken_), and fish and rabbit (PRP_fish_; PRP_rabbit_). The difference between PRP_deer_ and PRP_rabbit_ was over 20 nmol of trolox equivalent (Figure 1A). Secondly, the ability of promoting wound healing of these PRPs was also evaluated in cultured keratinocytes in vitro. The results showed that the best performer was PRP_deer_, which had a wound recovery rate of about 45.6% over the control group at 9.7%, followed by PRP_human_ and PRP_bovine_ (Figure 1B). The other PRP sources, however, were low (less than 33%) in wound-healing process. Application of VEGF served as a positive control here. Skin brightening efficacy is a very important metric when developing skincare products, and which is often evaluated by using melanin scavenging capacity. Here, we compared the melanin-reducing properties of different PRP samples in cultured melanocytes. PRP_deer_ showed the best reduction in melanin content (Figure 1C). The other sources of PRP did not show such an obvious reduction. Vitamin C served as a control to downregulate melanin content. In view of these functionalities, PRP_deer_ was chosen for further analysis.

To evaluate toxicity and identify the ideal concentration of PRP_deer_ in cells, MTT assay was conducted in HaCaT keratinocytes and melanocytes. Experimental results showed that in the PRP samples, taking PRP_deer_ as an example, significant cell death did not show when treated with PRP_deer_ from 0.5–50 mg/mL. A robust proliferative effect was found at 50 mg/mL PRP_deer_ in keratinocytes and 0.5 to 20 mg/mL in melanocytes (Figure 1D). High concentration of PRP_deer_, however, achieved a decrease in cell number, in accordance with the human source of PRP [22]. To avoid induction of cytotoxicity, the concentration of PRP_deer_ 1–15 mg/mL in this study was applied. The migration ability of PRP_deer_ in different concentrations was determined. The enhancement of wound healing did not show a significant increase at the lower concentrations of 1 and 2 mg/mL of PRP_deer_. However, there was a significant increase from 25% at 5 mg/mL to 65% at 15 mg/mL, which was better than that of the VEGF control, suggesting that PRP_deer_ should have excellent wound-healing ability (Figure 1E,F).

**Figure 2:**
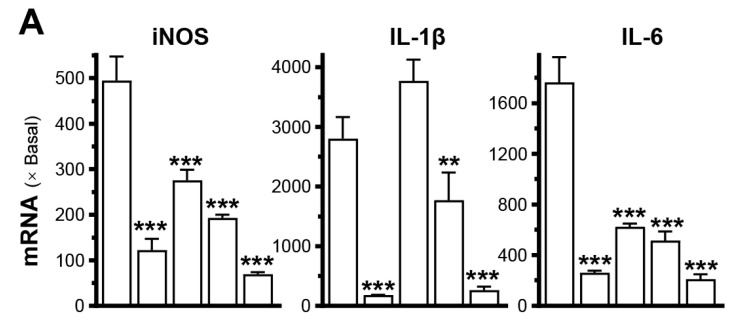

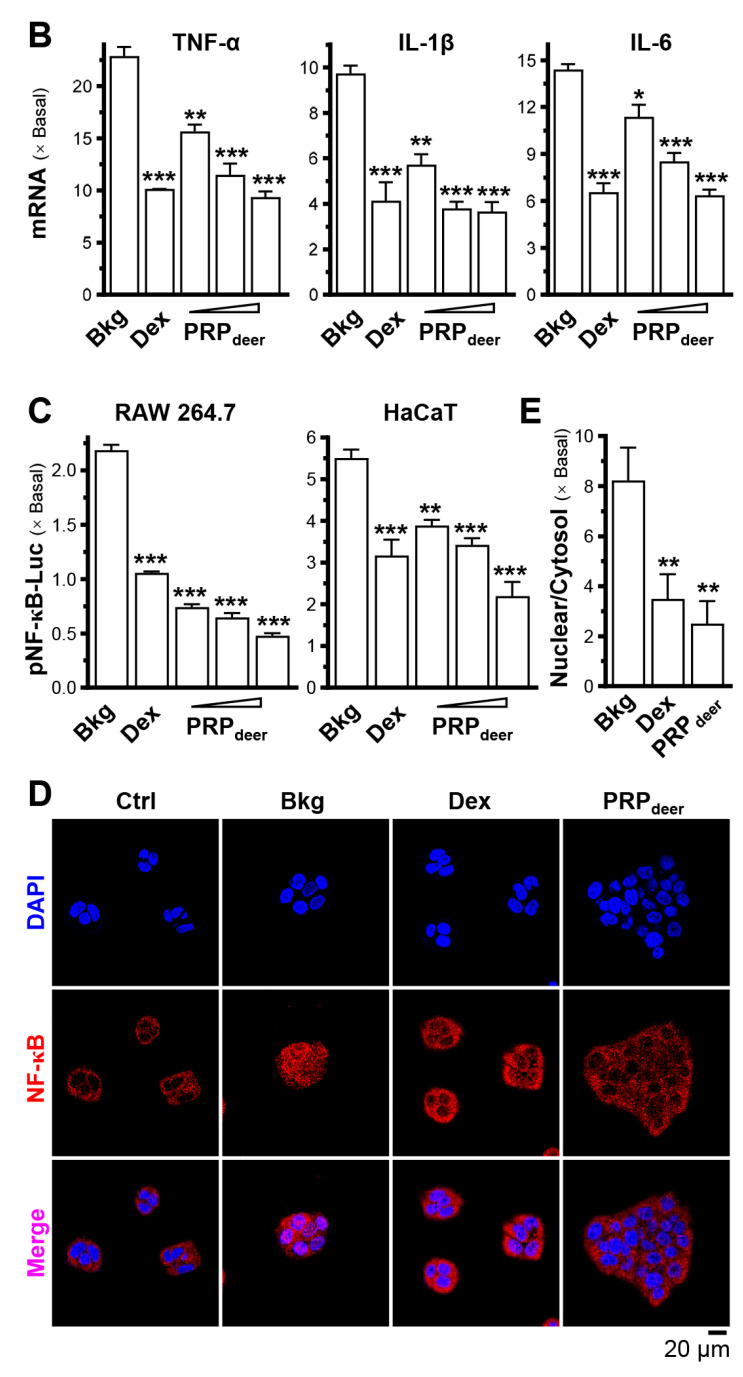
PRP_deer_ reduces the induced inflammatory responses. (**A**) The mRNA levels of iNOS, IL-1β, and IL-6 were measured in inflammatory activated cultured RAW264.7 cells after being pretreated with PRP_deer_ (1, 5, 10 mg/mL) for 4 h. Then, the exposure of LPS (100 ng/mL) was 20 h. Dexamethasone at 10 nM was a positive control. (**B**) The mRNA levels of TNF-α, IL-1β, and IL-6 in cultured HaCaT cells after 4 h of pretreatment of dexamethasone (10 nM) and PRP_deer_ (10 mg/mL), and 20 h of TNF-α (20 ng/mL) stimulation. (**C**) The pNF-κB-Luc plasmid was transfected into RAW264.7 or HaCaT cells for 4 h, followed by pretreating with PRP_deer_ (1, 5, 10 mg/mL) for 4 h, before the exposure to LPS (100 ng/mL) or TNF-α (20 ng/mL), respectively, for 20 h. Dexamethasone (10 nM) served as a control. (**D**) The protein level of NF-κB p65 in cytosolic and nuclear cells was measured in HaCaT keratinocytes, in representative confocal images. (**E**) The quantifications of intensity percentage in (**D**). The values are expressed in the fold of change to the normalized basal activity set at 1, in mean ± SEM., *n* = 8. * *p* < 0.05; ** *p* < 0.01; *** *p* < 0.001 compared with LPS- or TNF-α-treated groups (Bkg).

LPS-stimulated macrophages (RAW264.7 cells) were used as a model to mimic chronic inflammation. Treatment with LPS led to a significant increase in inflammatory markers’ expression, such as iNOS, IL-6, and IL-1β, ranging from 20 to 2000 times in cultured RAW264.7 cells (Figure 2A). Dexamethasone effectively suppressed the expression of these inflammatory markers in LPS-treated cultures. Three different concentrations of PRP_deer_ (0–10 mg/mL) were tested. Under the treatment of different concentrations of PRP_deer_, the concentrations of iNOS, IL-1β, and IL-6 markers were markedly suppressed in cultured LPS-stimulated macrophages (Figure 2A).

The effects of PRP_deer_ samples on keratinocytes were evaluated using TNF-α treatment. After TNF-α treatment, the mRNA levels of TNF-α, IL-1β, and IL-6 increased significantly by 10–23-fold in keratinocytes (Figure 2B). However, the treatment with PRP_deer_ markedly reduced these mRNA expressions in keratinocytes. The maximal suppression observed for TNF-α expression was approximately 70% (Figure 2B).

A DNA construct, pNF-κB-Luc, was used for plasmid transfection in both RAW264.7 and keratinocyte cell models. In RAW264.7 cells, LPS induced the luciferase signal, indicating promoter activity, by more than 2-fold, while TNF-α increased the signal by approximately 5.5-fold in HaCaT cells (Figure 2C). Following co-treatment with PRP_deer_, luciferase activity significantly decreased, showing reductions of 20% to 75%. Furthermore, the protein level of NF-κB p65 was evaluated in keratinocytes by immunostaining. When inflammation occurs, p65 protein migrates from the cytoplasm to the nucleus, so detection of the percentage of nuclear/cytosol of NF-κB p65 protein can reflect inflammation; the results revealed a significant decrease, showing about a 70% reduction after treatment with PRP_deer_ (Figure 2D,E).

### 3.2. PRP_deer_ Potentiates Skin Rejuvenation

**Figure 3:**
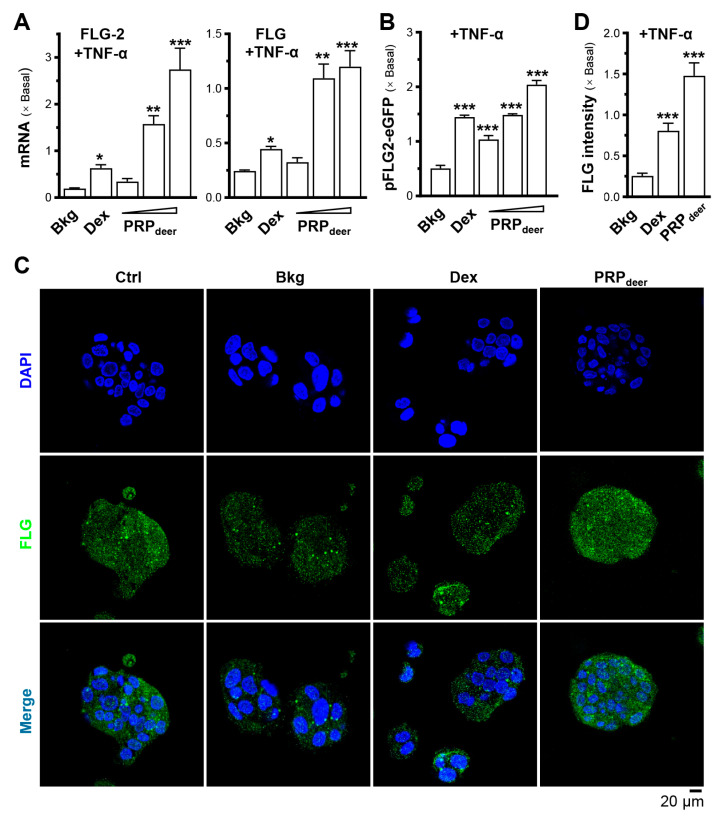
PRP_deer_ reverses the decrease in filaggrin and filaggrin-2 expressions caused by TNF-α by promoting moisturization and differentiation. The cultured HaCaT keratinocytes were pretreated with dexamethasone (10 nM), PRP_deer_ (1, 5, 10 mg/mL) for 4 h. Then, the exposure of TNF-α (20 ng/mL) was 20 h. (**A**) The mRNA levels of filaggrin (FLG) and filaggrin-2 (FLG-2) were evaluated by RT-PCR. (**B**) The pFLG2-eGFP plasmid was transfected into HaCaT cells for 4 h, followed by another 4 h pre-incubation of PRP_deer_ before 20 h stimulation of TNF-α. Dexamethasone was a control. (**C**) HaCaT keratinocytes were treated with TNF-α, dexamethasone, PRP_deer_ (10 mg/mL), as given in (**A**). The cultures were stained with FLG antibodies or DAPI. Representative fluorescence images are displayed. (**D**) The FLG intensity was quantified from (**C**). Values are normalized basal expression set at 1, in mean ± SEM., *n* = 5. * *p* < 0.05; ** *p* < 0.01; *** *p* < 0.001 compared with TNF-α-treated group (Bkg).

To assess the moisturizing effects of PRP_deer_ on skin keratinocytes, we evaluated its ability to regulate the mRNAs of filaggrin and filaggrin-2. Treatment with PRP_deer_ showed a significant, dose-dependent rise in both filaggrin and filaggrin-2, with filaggrin-2 exhibiting a notable sensitivity to the treatment, showing a maximum 2-fold increase in expression (Figure 3A). To demonstrate activated gene transcription, the filaggrin-2 promoter was tagged with an eGFP construct, i.e., pFLG2-eGFP. In keratinocytes transfected with pFLG2-eGFP, PRP_deer_ application enhanced the fluorescent signal, indicating increased promoter activity, with a robust 2-fold increase in pFLG2-eGFP fluorescence, surpassing the positive control of dexamethasone (Figure 3B). Filaggrin, a protein that is not associated with membranes and is in keratohyalin granules, was detected using immunochemical staining in cultured keratinocytes. Treatment with PRP_deer_ induced a significant increase in overall cellular staining for filaggrin, consistent with the mRNA and promoter assay results (Figure 3C,D).

**Figure 4:**
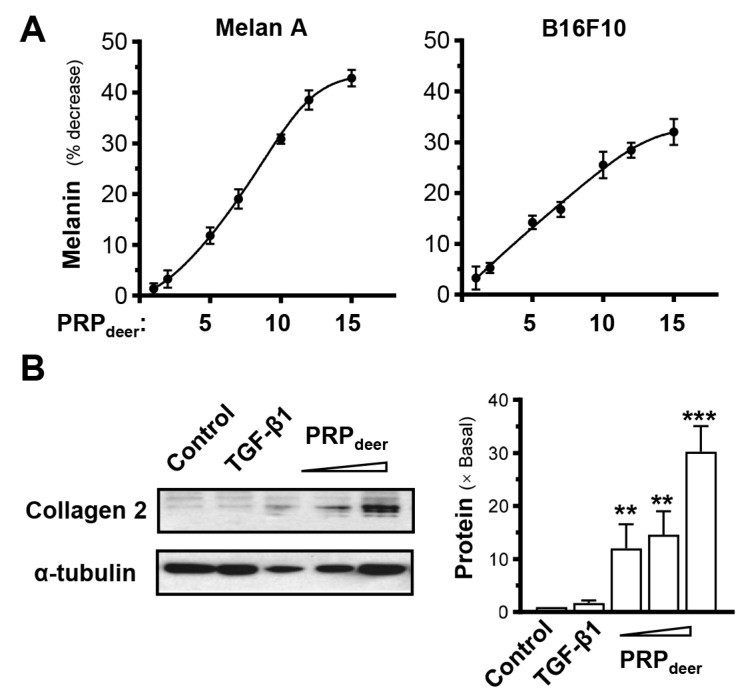
PRP_deer_ regulates the levels of melanin and collagen. (**A**) Cultured melanocytes (Melan-A) and melanoma (B16F10) were plated onto 6-well plates. PRP_deer_ (1 to 15 mg/mL) was administered for 48 h, and the melanin content was assessed. Vitamin C (10 μM, positive control) demonstrates an approximate 40% reduction. (**B**) Cultures were treated as in (A), and collagen 2 (~141 kDa) was evaluated by Western blot assays; α-tubulin (~55 kDa) served as internal control for quantification (**left** panel). TGF-β1 (10 ng/mL, positive control). The quantification was conducted by ImageJ software (2.1.4.5) (**right** panel). Values are expressed as the percentage of decrease in the control or the fold of change relative to normalized basal expression set at 1, in mean ± SEM., *n* = 5. ** *p* < 0.01; *** *p* < 0.001 compared with the control.

To investigate PRP_deer_ on melanogenesis, the productions of melanin in both Melan-A and B16F10 cells were conducted. The results showed that a higher concentration of PRP_deer_ had a stronger ability to inhibit melanin production (Figure 4A). When the PRP_deer_ sample concentration was 1 mg/mL, the inhibition rate was only around 2%, while when PRP_deer_ increased to 15 mg/mL, the melanin inhibition reached over 40% in Melan-A. The results were similar when the experiment was repeated on cultured B16F10 cells (Figure 4A). The inhibition of melanin by PRP_deer_ had an obvious dose-dependent relationship at ~10 mg/mL of PRP_deer_.

Collagen is a key factor in determining skin elasticity, and dermal fibroblasts are the primary cells responsible for collagen production in the skin. Therefore, the PRP_deer_ was applied onto cultured mouse dermal fibroblasts. By using an antibody recognizing collagen type II, the result showed that PRP_deer_ at a high dose could significantly induce collagen production by ~30-fold in cultured dermal fibroblasts (Figure 4B), indicating that PRP_deer_ could promote skin elasticity.

### 3.3. Proteomics Analysis of PRP_deer_ and PRP_human_

**Figure 5:**
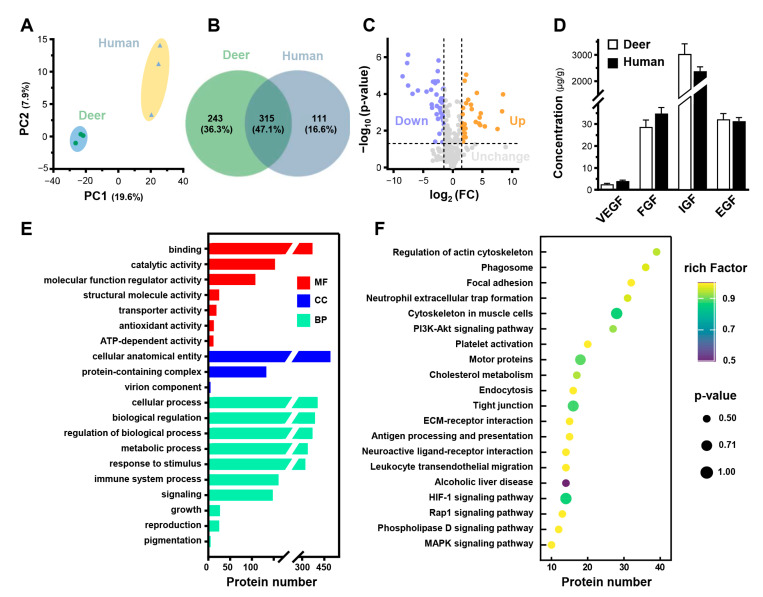
Proteomics analysis of PRP_deer_ and PRP_human_. (**A**) PCA scores for proteomic analysis of PRP_deer_ and PRP_human_. (**B**) Venn diagram showing the overlapping and unique proteins in PRP_deer_ and PRP_human_. (**C**) Volcano plot analysis of proteomic changes in PRP_deer_ and PRP_human_. (**D**) The amounts of VEGF, FGF, IGF, and EGF in PRP_deer_ and PRP_human_ were measured by ELISA. (**E**) GO analysis of proteins enriched in PRP_deer_. (**F**) KEGG displays the top 20 pathways analysis of PRP_deer_ proteins.

To evaluate the potential proteins of PRP_deer_ in wound healing, a non-labeling proteomic analysis was performed, using PRP_human_ protein as a control. Proteomic profiles were generated, and principal component analysis (PCA) was conducted to calculate scores for all samples. In the PCA plots, PRP_deer_ subjects were distinctly separated from PRP_human_ (Figure 5A). The three independent subgroups showed significant overlap, indicating good stability and reliability in sample measurement and data analysis. The Venn diagram illustrated the number of identified proteins with significant quantitative similarities and differences among the three groups (Figure 5B). A total of 558 proteins were identified in PRP_deer_, while 426 proteins were identified in PRP_human_. Of these, 315 proteins were shared between the two groups, indicating a substantial overlap of 47.1%, which supports the robustness of the proteomic maps. Additionally, a volcano plot of overlapping proteins was generated (Figure 5C). A total of 72 significant differential proteins were identified, representing 12.9% of all detected proteins, with 29 proteins showing significant upregulation and 43 proteins showing significant downregulation.

FGF, VEGF, EGF, and IGF are common ingredients in mammalian blood, as well as in PRP, and are well known to promote wound healing. The proteomic of PRP_deer_ indicated the existence of the fragments of these proteins (Table S1). Thus, the existence of growth factors in the prepared PRP from deer and humans was confirmed. Both PRP_deer_ and PRP_human_ contained similar amounts of VEGF, FGF, IGF, and EGF (Figure 5D). IGF showed the largest concentration in PRP_deer_ at 3036 µg/g, slightly higher than that of PRP_human_ at 2380 µg/g. Also, PRP_deer_ and PRP_human_ showed high levels of EGF and FGF, both over 30 µg/g. VEGF was the lowest among these growth factors with a concentration of less than 5 µg/g.

To further investigate the functions of proteins in PRP_deer_, GO and KEGG pathway enrichment analyses were performed. GO analysis assessed cellular components (CCs), molecular functions (MFs), and biological processes (BPs) for PRP_deer_ (Figure 5E). The cellular component analysis indicated that 88.2% of the identified proteins were associated with cellular anatomical entities. Molecular function analysis revealed that binding (66.1%) was the predominant category for PRP_deer_ proteins. Additionally, around 19, 13, and 12 proteins were linked to transporter activity, antioxidant activity, and ATP-dependent activity, respectively. Biological process analysis highlighted an enrichment of proteins involved in cellular processes (72.6%), metabolic processes (69.4%), and biological regulation (65.6%). Other notable categories included immune system processes (28.9%), cell proliferation (4.8%), and growth (4.5%). These proteins may be closely related to the antioxidant and wound-healing capabilities of PRP_deer_. A comprehensive list of the identified proteins can be found in Table S1. Subsequently, KEGG analysis identified the top 20 pathways associated with PRP_deer_ proteins. Key pathways included “Focal adhesion”, “PI3K-Akt signaling pathway”, “Platelet activation”, “HIF-1 signaling pathway”, and “MAPK signaling pathway”, all of which play significant roles in wound healing (Figure 5F).

### 3.4. Metabolomics Analysis of PRP_deer_ and PRP_human_

**Figure 6:**
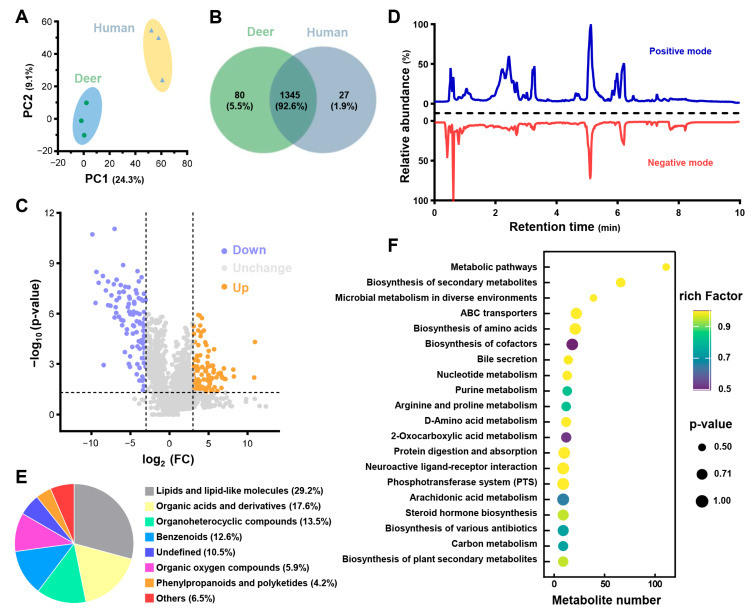
Metabolomics analysis of PRP_deer_ and PRP_human_. (**A**) PCA scores for metabolomics analysis of PRP_deer_ and PRP_human_. (**B**) Venn diagram showing the overlapping and unique metabolites in PRP_deer_ and PRP_human_. (**C**) Volcano plot analysis of metabolite changes in PRP_deer_ and PRP_human_. (**D**) Extracted ion chromatogram of PRP_deer_ metabolomic. (**E**) Chemical categories of metabolites in PRP_deer_. (**F**) KEGG displays the top 20 pathways analysis of PRP_deer_ metabolites.

Global metabolic profiles of PRP_deer_ and PRP_human_ were analyzed using UHPLC-MS/MS, and PCA analysis revealed a clear distinction between the two groups, confirming the reliability of the metabolomics data (Figure 6A). After the annotation, 1425 metabolites were identified in PRP_deer_ and 1372 in PRP_human_ (Figure 6B). Notably, 1345 metabolites were common to both groups, indicating a significant overlap of 92.6%, which supports the robustness of the metabolite maps. To further investigate the differences between PRP_deer_ and PRP_human_, statistical analysis was performed on the metabolic profiles, resulting in a volcano plot shown in Figure 6C. This analysis identified 209 significant differential molecules, representing 14.7% of all detected metabolites, with 101 showing significant upregulation and 108 downregulation. The extracted ion chromatogram (XIC) revealed thousands of mass spectral features primarily eluting in both positive and negative modes for PRP_deer_ within the first 10 min (Figure 6D). The metabolites were classified based on their chemical properties (Figure 6E), with the majority being lipids and lipid-like molecules (29.2%), followed by organic acids and derivatives (17.6%), organo-heterocyclic components (13.5%), and benzenoids (12.6%). A comprehensive list of the identified metabolites can be found in Table S2.

To further explore the metabolic disturbances in PRP_deer_, pathway enrichment analysis was conducted, with the top 20 pathways illustrated in Figure 6F. Most pathways were linked to metabolic processes, biosynthesis of secondary metabolites, and microbial metabolism in various environments. Additionally, 39 metabolites were associated with the biosynthesis of an amino acid’s pathway, which may be relevant to wound healing.

The abundance of different proteins and metabolites in PRP_deer_ varied greatly. The molecules related to skin rejuvenation are proposed for quantifying control on the isolated PRP. As listed in Table 1, collagen type VI α3 chain and dexpanthenol, playing important roles in skin rejuvenation, are widely expressed in PRP_deer_. In addition, PRP_deer_ contains excellent antioxidants, such as hemopexin, niacinamide, and glutathione oxidized. The PRP_deer_ also contains a number of proteins and metabolites associated with wound healing, such as Ig-like domain-containing protein, apoptosis inhibitor, vitronectin, hydroxy arginine, 6-ketoprostaglandin E1, palmitic acid, and AMP; these metabolites are important in promoting cell migration. PRP_deer_ also contains a variety of growth factors, such as IGF and EGF, which may be the main proteins involved in promoting angiogenesis, while the pathways related to angiogenesis in PRP_deer_ include PI3K-Akt and the HIF-1 signaling pathway. PRP_deer_ contains inflammatory-responsive molecules, such as complement C3, alpha-2-macroglobulin, plasma kallikrein, arachidonoyl serotonin, and docosatetraenoic acid. Arachidonoyl serotonin, formed by the conjugation of arachidonic acid and serotonin, and 6-ketoprostaglandin E1a produced during the metabolism of arachidonic acid are enriched in PRP_deer_. Thus, arachidonic acid could be a potential active substance in the promotion of wound healing by PRP; however, this remains to be verified at the molecular level.

### 3.5. Safety Parameters of PRP_deer_

**Figure 7:**
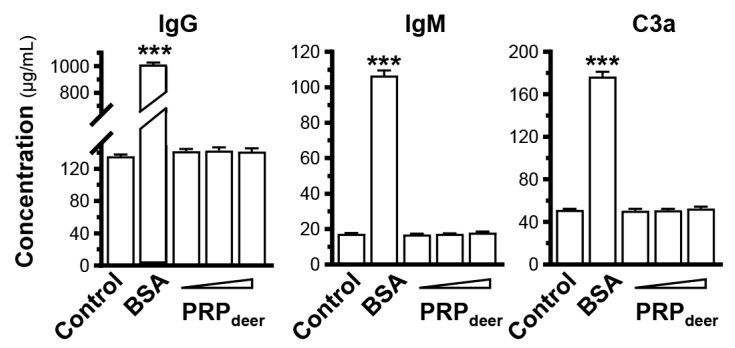
Immunostimulatory analysis of PRP_deer_. After treated with bovine serum albumin (BSA, 1 mg/kg) or PRP_deer_ (50, 100, and 200 mg/kg) for 1 month with weekly intravenous injection, the amounts of IgG, IgM, and C3a in the serum of mice were measured by ELISA. The values are in mean ± SEM., *n* = 10. *** *p* < 0.001 compared with control.

To test the safety of PRP_deer_, the mice were injected by intravenous of the PRP. After a month of weekly injections, the concentrations of IgG, IgM, and C3a in the serum showed no significant differences among these three PRP groups. In comparison, the BSA group exhibited a notable increase when contrasted with the control group (Figure 7). Thus, the doses of PRP_deer_ showed no immune activation even at high doses. The Ames Bacterial Reverse Mutation Test was used to test the mutagenic potential of PRP_deer_. In the test, TA97a, TA98, TA100, TA102, and TA1535—five *Salmonella typhimurium* strains—were employed. The number of colonies was calculated to determine the genotoxicity of PRP_deer_. Under the experimental conditions, compared with the solvent control, among the 20 samples of PRP_deer_ that were tested, the number of revertant colonies had no significant difference (Table 2). Thus, PRP_deer_ had no mutagenic effect on *Salmonella typhimurium*.

## 4. Discussion

Regenerative medicine is a developing area focused on replacing or restoring damaged tissues or diseased tissues and organs using biological materials and engineering approaches [2]. PRP has become a promising therapeutic intervention in regenerative medicine because of its capacity to influence various cellular processes related to tissue repair and regeneration [10,15]. However, the availability of human blood donors may be limited in certain regions or populations, and human-derived PRP may be more expensive to prepare and require more stringent regulatory oversight. As a result, animals have become a popular source of PRP due to their ready availability and ability to provide large quantities of blood for preparation, which is particularly useful in research studies where large quantities of PRP are required for experimental purposes. It is important to note that PRP derived from different animal sources can vary greatly. The efficacy of PRP preparations is intrinsically linked to interspecies variations in hematological parameters, particularly the platelet-to-erythrocyte volume ratio. Empirical evidence confirms that centrifugal force and duration critically modulate PRP quality metrics, including mean platelet volume (MPV), platelet recovery efficiency, and functional integrity [28]. Comparative studies in caprine and ovine models demonstrate that gradient centrifugation protocols employing reduced centrifugal forces (e.g., 200× g) with extended durations (15 min) enhance the platelet yield by 30–40% while concurrently elevating MPV [29]. This phenomenon is attributed to the preferential sedimentation of erythrocytes under low-speed conditions, thereby minimizing platelet loss during phase separation.

The choice of animal species affects the composition and activity of the PRP products, as well as the potential risks associated with that, including the risk of transmitting infectious diseases and the risk of immunogenic reactions. In a study, the authors extracted PRP from humans, rats, and goats, using rats as a model to compare the effects of PRP from these three different species on bone regeneration. The results showed that PRP derived from both rats and goats had no significant effect on bone formation. However, human PRP was found to improve the initial osteogenic response of human bone grafts [30]. In contrast, another study reported that PRP derived from dogs demonstrated bone regeneration efficacy [31]. The studies suggest that the differences in functions among different animals may be related to variations in platelet content and types of growth factors in their PRPs. The average platelet count in rats is significantly higher than that in humans, and parallelly the average platelet volume in rats is significantly lower. Given the low platelet volume in rats and that growth factors are stored in the alpha granules of platelets, we can expect varied concentrations in growth factors between rat and human platelets. Quantitative analyses of growth factors in PRP prepared from rats, pigs, goats, and humans show that human PRP contains higher concentrations of TGF-β1 and PDGF αα, ββ, and αβ30 per platelet, as compared to rats, pigs, and goats [32,33]. Our results indicated that both deer PRP and human PRP contained similar amounts of VEGF, FGF, IGF, and EGF. It can be inferred that after platelet activation, deer PRP could release more growth factors compared to rat, pig, and goat PRPs, resulting in the more pronounced effect of deer PRP.

Thus, selecting the right source of PRP for applications is essential to guarantee the product’s safety and effectiveness. To address this issue, we have conducted a study to screen several common animal types of PRP and evaluate them in terms of antioxidant activity, wound-healing ability, and inhibition of inflammation and melanogenesis. Our results indicated that the deer blood-derived PRP excelled in all indicators. Additionally, we performed genotoxicity testing and found that deer blood was non-toxic and non-mutagenic, suggesting that deer blood may be an ideal source of animal-derived PRP for certain applications. In addition, the farming of deer is very popular in China. The antler base from male *C. nippon* or *C. elaphus* has been extensively utilized in Chinese medicine to treat various ailments. Over 260,000 deer are raised mainly for velvet antler production in China. Blood withdrawal is a normal procedure to stimulate the growth of antlers, and this blood collection could be a means of recycling the waste products during deer farming.

For a better medical application, PRP_deer_ is necessary to be standardized by using established biomarkers before assessing the skincare-related biological activities of PRP_deer_. When selecting protein biomarkers in PRP, several factors must be considered, including their relevance to the target tissue or condition, their specificity and sensitivity, and their stability and reproducibility [34,35]. Here, we measured the proteins and metabolites of PRP_deer_. The proteins, including vitronectin, fibrinogen alpha chain, and plasma kallikrein, having important roles in the maintenance of platelet function and wound healing, were highly expressed in PRP_deer_, and therefore, these proteins could be ideal candidates as biomarkers for quality control. Based on the same criteria, the non-protein components, e.g., arachidonic acid, glutathione oxidized, and palmitic acid, significantly enriched in PRP_deer_ and having positive effects on wound healing, could be another biomarker. In addition, the high contents of growth factors, e.g., FGF, IGF, VEGF, and EGF, in PRP_deer_, could also be included as biomarkers for quality control. A study compared the protein composition of human platelet-poor plasma and human platelet lysate, revealing that the majority of the proteins specific to human platelet lysate are implicated in the stress response and wound healing [36]. This finding provides a reference for our subsequent research on standard markers from the deer source.

Our study showed that PRP_deer_ may enhance angiogenesis and encourage both cell proliferation and migration, and have anti-inflammatory effects, which can be particularly beneficial in the context of tissue injury and repair. The underlying mechanisms of therapeutic effects of PRP on tissue repair and regeneration are complex and should be multi-factorial. PRP is rich in growth factors that are known to promote angiogenesis as well as stimulate cell proliferation and migration [11]. Furthermore, PRP can boost the accumulation of extracellular matrix components, like collagen and fibronectin, which are crucial for tissue repair and regeneration [10]. Our proteomics analysis showed that many signaling proteins relating to the above functions were being enriched within PRP_deer_, including MAPK signaling and PI3K/Akt signaling. Thus, animal-derived PRP is a promising alternative to human-derived PRP in regenerative therapy and tissue fabrication. However, the animal species are critical to guaranteeing the safety and effectiveness of the PRP product. Our study suggests that deer blood may be an ideal source of animal-derived PRP for clinical applications.

## 5. Conclusions

Here, we provide compelling evidence of the superior skincare properties of PRP_deer_, demonstrating its efficacy in rejuvenating skin, as well as surpassing the functionalities and contents of growth factors of other animal sources. Additionally, we conducted a thorough analysis of the protein composition and safety of PRP_deer_. These results suggest promising applications for PRP_deer_ in both cosmetics and/or medical treatments.

## Figures and Tables

**Table 1:** The main proteins and metabolites related to wound healing and skin rejuvenation in PRP_deer_.

Function	Molecules		Biological Process ^c^
	Proteins ^a^	Metabolites ^b^	
Skin rejuvenation	COL6A3	Dexpanthenol	Collagen trimer
	Hemopexin	Niacinamide, Glutathione oxidized	Antioxidant
Wound healing	Ig-like domain-containing protein, Apoptosis inhibitor, Vitronectin	Hydroxyarginine, 6-Ketoprostaglandin E1, Palmitic acid, AMP	Cell migration
	Fibronectin, IGF, EGF	Arachidonic acid	Angiogenesis
	Complement C3, Alpha-2-macroglobulin, Plasma kallikrein	Arachidonoylserotonin, Docosatetraenoic acid	Anti-inflammatory

^a^ The proteins from the results of proteomics list in Table S1; ^b^ the metabolites from the results of metabolomics list in Table S2; ^c^ biological process analysis based on the results of KEGG analysis of PRP_deer_ proteins and metabolites.

**Table 2:** Genotoxicity analysis of PRP_deer_ by bacterial reverse mutation Ames test.

Strains ^a^	Treatment	Number of Colonies		Genotoxic
		No metabolic Activation	Metabolic Activation	
TA97a	Positive control	1052.7 ± 31.7 ^b^	1145.7 ± 12.3	+ ^c^
	Solvent Group	163.7 ± 2.5	173.7 ± 4.9	−
	Untreated Group	162.3 ± 1.5	172.7 ± 2.1	−
	Test Group	162.7 ± 3.5	169.7 ± 3.5	−
TA98	Positive control	455.7 ± 36.2	557.0 ± 25.9	+
	Solvent Group	44.3 ± 1.5	44.7 ± 1.5	−
	Untreated Group	44.7 ± 2.5	44.7 ± 2.5	−
	Test Group	44.0 ± 1.0	44.3 ± 1.5	−
TA100	Positive control	1057.0 ± 28.5	1142.3 ± 15.8	+
	Solvent Group	164.7 ± 4.0	173.0 ± 3.0	−
	Untreated Group	162.7 ± 1.5	171.3 ± 3.2	−
	Test Group	142.7 ± 2.5	171.3 ± 4.2	−
TA102	Positive control	958.7 ± 26.1	1058.3 ± 36.6	+
	Solvent Group	314.3 ± 3.5	325.0 ± 3.0	−
	Untreated Group	313.7 ± 5.7	323.3 ± 3.2	−
	Test Group	312.3 ± 3.2	324.9 ± 4.2	−
TA1535	Positive control	95.3 ± 2.5	356.7 ± 28.4	+
	Solvent Group	27.0 ± 1	28.7 ± 2.1	−
	Untreated Group	28.0 ± 2.6	28.3 ± 3.1	−
	Test Group	28.3 ± 2.1	28.0 ± 3.0	−

^a^ *S. typhimurium* strains (TA97a, TA98, TA100, TA102, and TA1535) were used; ^b^ Values are in mean ± SEM., *n* = 20; ^c^ “+” indicates genotoxicity, “−” indicates non-genotoxicity.

## Data Availability

The original contributions presented in this study are included in the article/Supplementary Materials. Further inquiries can be directed to the corresponding author.

## Supplementary Materials

The following supporting information can be downloaded at https://www.mdpi.com/article/10.3390/cells14070526/s1. Table S1: the full list of the obtained proteins of PRPdeer; Table S2: the full list of the obtained metabolites of PRPdeer.
